# Case Report: Popliteal artery entrapment syndrome as a cause of deep vein thrombosis and subsequent popliteal artery occlusion

**DOI:** 10.3389/fsurg.2024.1384331

**Published:** 2024-05-06

**Authors:** Sangho Lee, Deokbi Hwang, Woo-Sung Yun, Seung Huh, Hyung-Kee Kim

**Affiliations:** ^1^ Division of Vascular and Endovascular Surgery, Department of Surgery, Kyungpook National University Hospital, School of Medicine, Kyungpook National University, Daegu, Republic of Korea; ^2^ Division of Vascular and Endovascular Surgery, Department of Surgery, Kyungpook National University Chilgok Hospital, School of Medicine, Kyungpook National University, Daegu, Republic of Korea

**Keywords:** case report, popliteal artery entrapment syndrome, popliteal vein, deep vein thrombosis, gastrocnemius muscle

## Abstract

**Background:**

Popliteal artery entrapment syndrome (PAES) is a relatively rare cause of arterial insufficiency in young and physically active individuals; however, deep vein thrombosis (DVT) can develop in association with PAES.

**Case report:**

A 47-year-old man presented with a 6-day history of left leg swelling and discomfort which was diagnosed as DVT extending to the distal femoral vein and pulmonary embolism on computed tomography (CT). PAES was not suspected at this time, and the patient was administered anticoagulants for 1 year. Two years after the DVT diagnosis, the patient developed sudden-onset left calf claudication for 1 week. Repeat CT angiography showed popliteal artery (PA) occlusion caused by PA displacement from an abnormally lateral insertion of the medial gastrocnemius head. A retrospective review of the initial CT scan confirmed this, as well as compression of the popliteal vein between the displaced medial head and the normal lateral head of the gastrocnemius. The patient eventually underwent myotomy and resection of the PA with an interposition graft.

**Conclusion:**

This case underscores the potential of PAES as a rare etiology of DVT, emphasizing the importance of considering it in the differential diagnosis of DVT in younger patients lacking common predisposing factors.

## 1 Introduction

Popliteal artery entrapment syndrome (PAES) is a rare vascular disorder that involves compression of the popliteal artery (PA) from the surrounding musculotendinous structures (1). PAES can be anatomical or functional—anatomical PAES results from PA compression or entrapment occurring due to anatomical anomalies that are often present at birth and developed over time, whereas in functional PAES, the compression is not due to a fixed anatomical anomaly but rather due to dynamic factors, such as muscle contraction during physical activity (1).

Although the incidence of functional PAES is not well characterized, the most common symptoms are intermittent claudication and pain in the feet and calves after exercise due to external compression of a normal artery. In anatomic PAES, the claudication could result from stenosis or occlusion of an injured PA by repeated trauma (2, 3). However, other clinical manifestations, such as chronic pain, acute limb ischemia, and pulsating mass due to aneurysmal changes in the PA, have also been reported in the literature (3, 4). Additionally, a few cases of deep vein thrombosis (DVT) have been reported in association with PAES (5). Since both DVT and subsequent PA occlusion are rarely associated with PAES, clinical suspicion is crucial, especially in young patients without precipitating factors for DVT.

In this report, we present a case of PAES complicated by DVT and subsequent PA occlusion occurring 2 years later. The patient was ultimately managed surgically with myotomy and interposition graft for the arterial occlusion.

## 2 Case description

A 47-year-old man presented with a 6-day history of left leg swelling and discomfort. He reported a history of hypertension for 5 years and had undergone gamma knife radiosurgery for an intracranial dural arteriovenous fistula 4 years ago. The patient denied any history of precipitating factors for DVT, including recent trauma, surgery, or immobilization.

**Figure 1:**
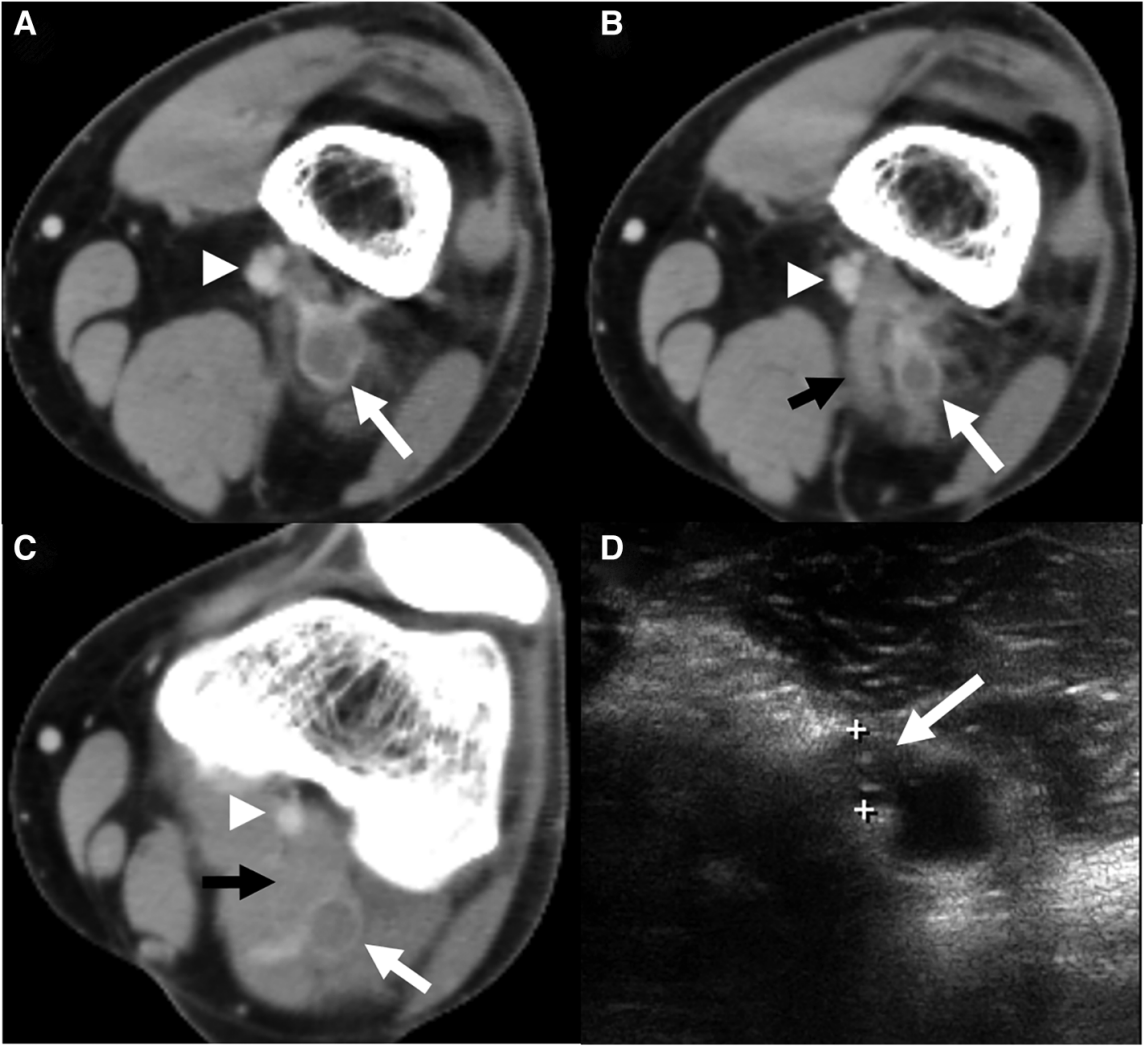
A computed tomography (CT) scan was conducted at the index visit. (**A**) Axial CT section revealed deep vein thrombosis (*white arrow*) of the popliteal vein (PV) with a patent popliteal artery (*arrowhead*). (**B**) Axial CT imaging of a point 1 cm distal showed the same findings with an abnormally inserted medial head of gastrocnemius muscle (*black arrow*) and thrombosed popliteal vein with most stenosis. (**C**) Axial CT of the mid portion of the popliteal fossa demonstrated thrombosed PV (*white arrow*) between both heads of the gastrocnemius muscle. (**D**) Duplex ultrasonography 1 year after anticoagulation demonstrated partial recanalization of the popliteal vein with residual hyperechoic fibrotic material (*white arrow*).

Duplex ultrasonography (DUS) conducted at the time of presentation revealed DVT extending to the distal femoral vein; this was confirmed in the subsequent contrast-enhanced computed tomography (CT), which also revealed pulmonary embolism in the right segmental pulmonary artery (Figure 1). Laboratory findings at initial presentation did not show any abnormalities (hemoglobin, 15.1 g/dl; platelet count, 180 K/µl, prothrombin time, 10.9 s; activated partial thromboplastin time, 26.2 s). Additionally, laboratory tests for hypercoagulability revealed normal values for natural anticoagulants, such as protein C, S, and antithrombin III; factor V Leiden, prothrombin, and JAK2 V617F mutations were not found. Accordingly, the patient was diagnosed with idiopathic venous thromboembolism; anticoagulation therapy with rivaroxaban was administered for 1 year, with a dosage of 20 mg for the initial 6 months and 10 mg for the subsequent 6 months for extended therapy, which was eventually discontinued. At this time, the follow-up DUS demonstrated partial recanalization of the popliteal vein (PV) with residual hyperechoic fibrotic material (Figure 1D). The waveform of PV venous flow showed continuous waveform and the axial reflux of PV was also noted. The patient complained of swelling in the left calf worsening in the afternoon or with exercise.

**Figure 2:**
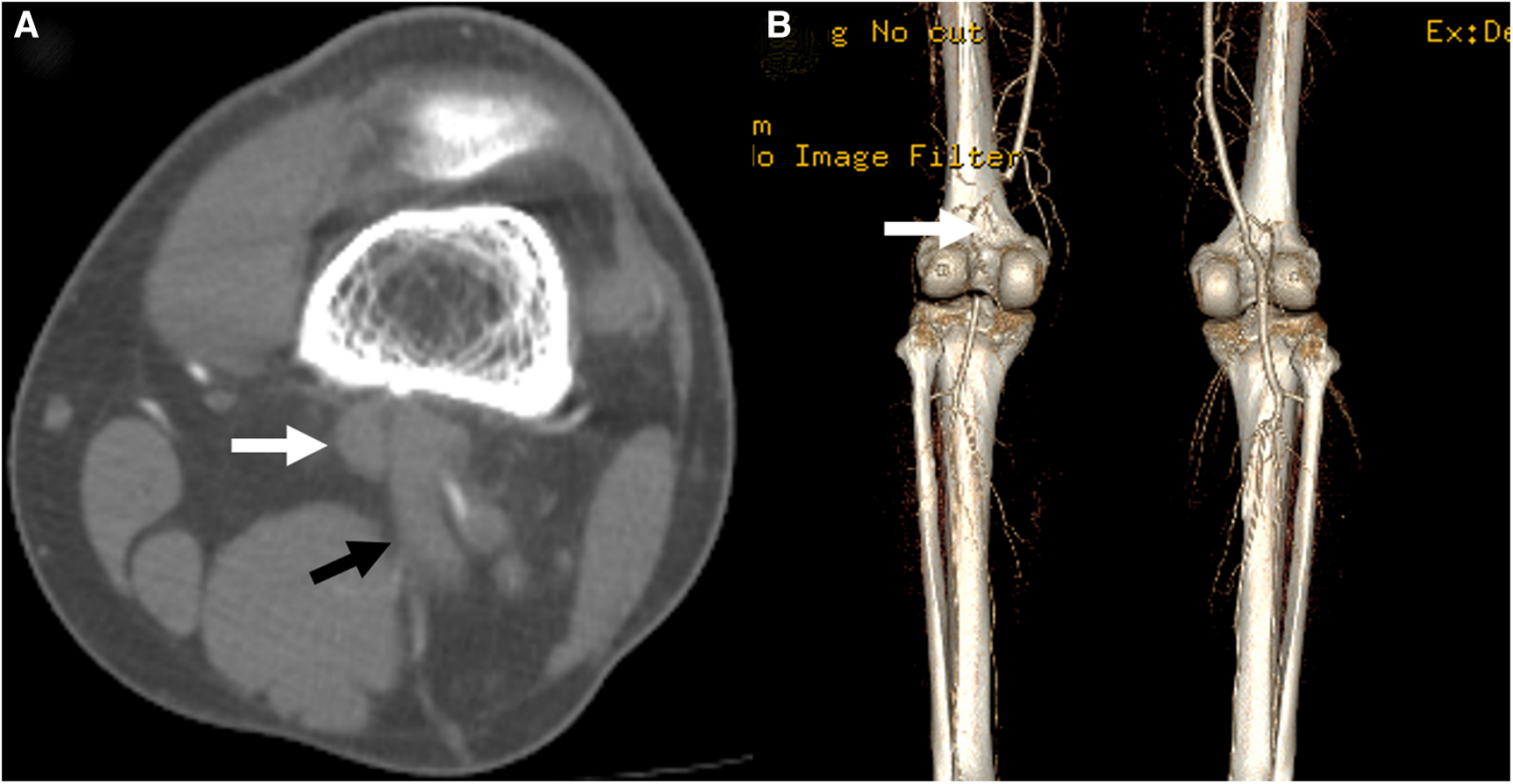
A computed tomography scan conducted at the time of the second presentation showed occlusion of the popliteal artery (*white arrow*) in the axial (**A**) and 3-dimensional reconstruction (**B**, posterior view) images. The anomalous insertion of the medial head of the gastrocnemius muscle between the popliteal artery and vein was also seen (*black arrow*).

Two years after this event, the patient again visited our outpatient clinic with sudden-onset claudication in the left calf for 1 week. On physical examination, the left ankle pulse was not palpable compared to a normal pulse in the right ankle [the ankle-brachial index (ABI) decreased to 0.62 on the left ankle]. Repeat DUS revealed occlusion of the PA with distal embolization to the dorsalis pedis artery. Subsequent CT angiography (CTA) showed occlusion of PA caused by type 2 PAES, i.e., medial deviation of the PA due to abnormal lateral attachment of the medial head of the gastrocnemius muscle between PA and PV (Figure 2) (6). A careful review of the CT conducted during the previous DVT event also confirmed this abnormal gastrocnemius insertion, and PV was thought to be compressed by both heads of the gastrocnemius muscle (Figure 1C).

**Figure 3:**
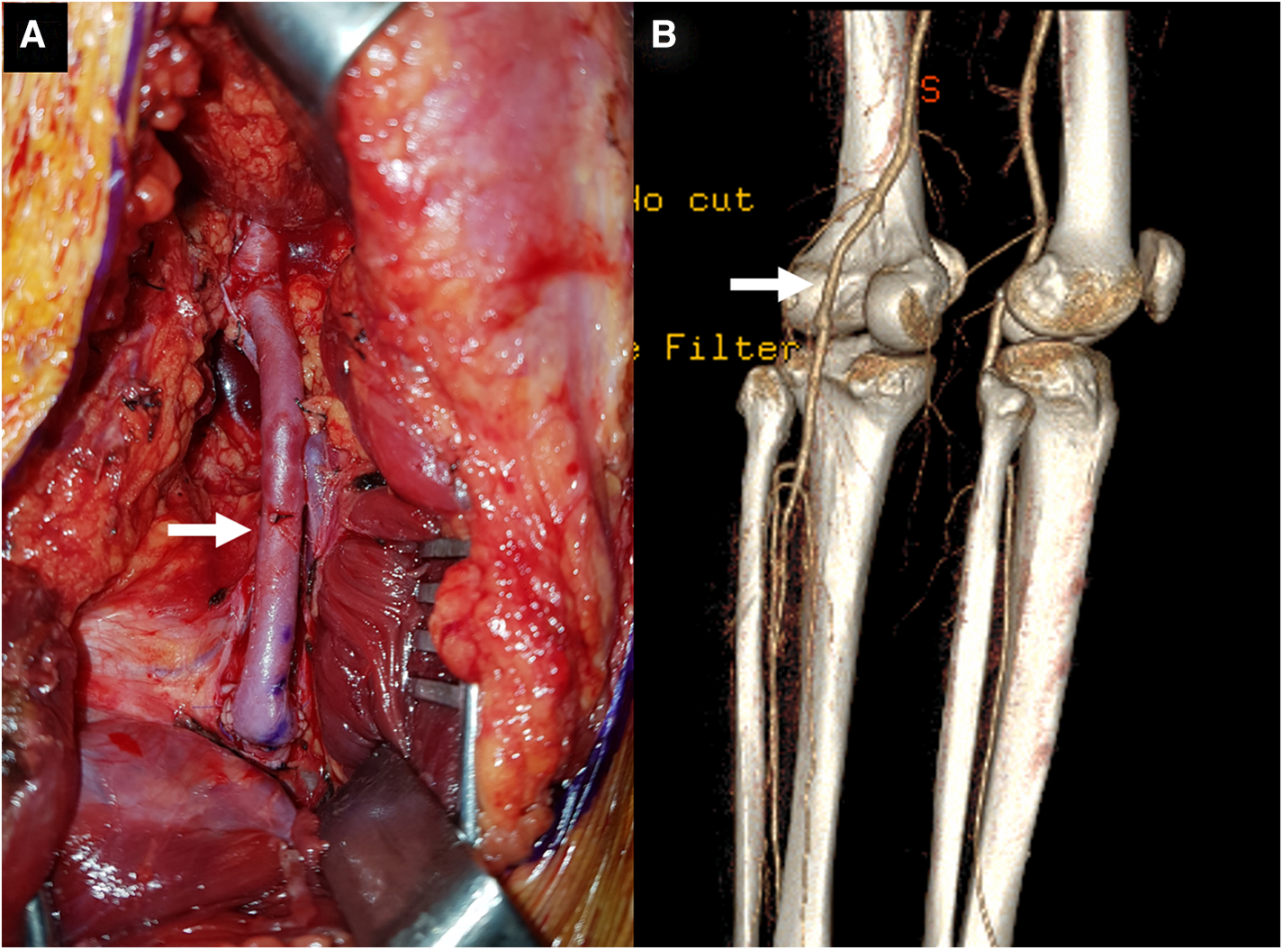
(**A**) The operative photograph showing a patent interposition graft (*arrow*) made using a reversed great saphenous vein at the distal thigh. (**B**) The follow-up computed tomography angiography showed a sufficiently patent graft (*arrow*) (posterior view).

**Figure 4:**
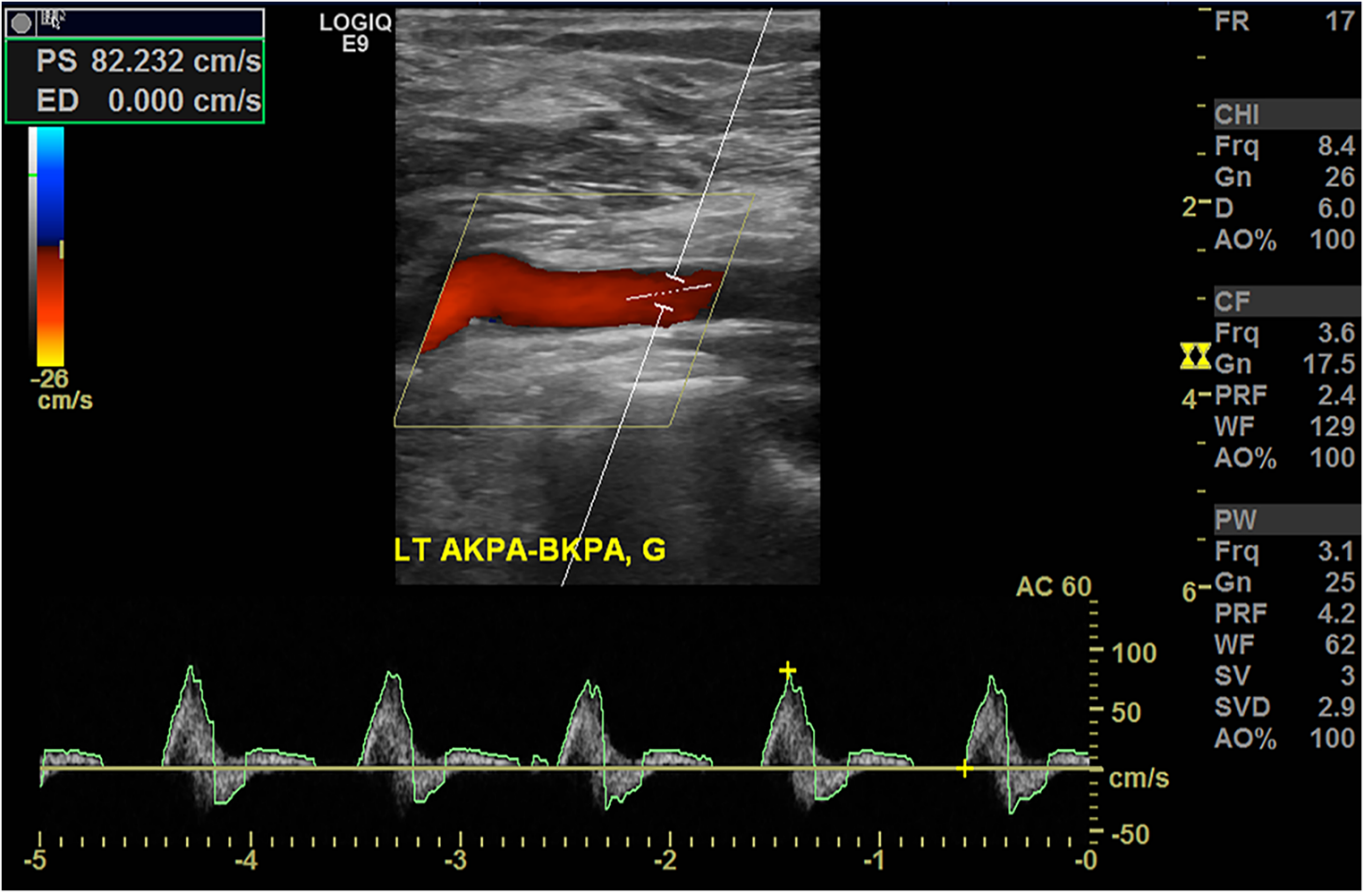
Duplex ultrasonography conducted 5 years after the operation demonstrating a patent interposition graft without stenosis.

Consequently, the patient was diagnosed with PAES with DVT and subsequent PA occlusion, for which he underwent surgical correction three days after presenting with PA occlusion. A myotomy of the medial head of the gastrocnemius was performed using a posterior S-shaped incision. Next, the injured part of the PA was resected and interposition grafting with the great saphenous vein harvested from the medial thigh was done (Figure 3A). The PV was not explored because the patient did not exhibit severe symptoms associated with chronic venous insufficiency. The postoperative course was uneventful, and the follow-up ABI before discharge was normalized to 1.10. The follow-up CTA before discharge also demonstrated a patent interposition graft with a typical course of PA (Figure 3B), so the patient was discharged a week after the operation. The patient received aspirin 100 mg monotherapy indefinitely following interposition grafting, and anticoagulation therapy was not restarted. During the subsequent 6 years of follow-up, he remained asymptomatic with a patent interposition graft which was monitored using DUS (Figure 4).

This study, serving as a care report, adhered to the CARE (CAse REport) guidelines. This study was approved by the institutional review board; informed consent was obtained from the patient (approval number: 2024-01-0033).

## 3 Discussion

DVT associated with PAES is a rare clinical presentation. The first case of PA and PV entrapment was reported in 1967 (7). At present, approximately 10%–15% of PAES cases are believed to involve the PV (5). While the most common cause of PV entrapment is an anomaly of the medial head of gastrocnemius (5, 8), other causative factors, including bone tumor, cysts within the popliteal fossa, PA aneurysm, and anomalous course of the nerve and short saphenous vein, have been reported (9–11). Even in patients without definite anatomic anomalies around the popliteal vessels, PV entrapment can be caused by hypertrophy of the gastrocnemius and popliteus muscles, similar to functional PAES (12).

In the standard classification system for PAES, type 1–4 PAES represent the isolated entrapment of PA due to the medial head or aberrant slip of the gastrocnemius and plantaris muscles (6, 13); any PV entrapment along with the PA is classified as a type 5 anomaly (1). Furthermore, functional PAES due to hypertrophy of surrounding muscles in the absence of anatomical anomalies of surrounding musculotendinous structures is classified as type 6. Our patient had type 2 PAES, which involves medial deviation of the PA due to abnormally lateral attachment of the medial gastrocnemius head between the PA and PV. In this case, the PV was not exactly trapped by the medial gastrocnemius head but was rather compressed between the two heads of the gastrocnemius (Figure 1) because of the abnormally lateral course of the medial head. A careful review of the CT scan conducted at the time of the index presentation demonstrated a stenotic segment within the thrombosed PV. Additionally, the patient had negative results for hypercoagulability; therefore, we presume that the abnormally inserted medial head of the gastrocnemius and compression between medial and lateral heads may have caused DVT in this patient.

The treatment of DVT associated with PAES is determined by the severity of DVT and nature of the presenting symptoms. Patients with thrombosis should be treated with anticoagulants, and if indicated like iliofemoral DVT and severe symptoms, catheter-directed thrombolysis may be considered (14). In patients with concomitant PA entrapment, surgical decompression and thrombectomy with or without popliteal vein reconstruction may be contemplated (14).

In our case, PAES was not suspected as the cause of DVT at initial presentation and the proximal extent of DVT was located in the distal femoral vein; therefore, only anticoagulant therapy was administered to him for a year. However, the patient developed PA occlusion associated with PAES 1 year after discontinuing the anticoagulants, for which he eventually underwent PA resection with an interposition graft. In a review of the index CT scan conducted at the time of the DVT event, the PA did not show any stenotic changes, thrombus formation, aneurysmal changes, or distal embolization to the tibial arteries. Therefore, if PAES is suspected as the cause of DVT in this patient, a less invasive operation, such as simple myotomy without PA reconstruction, may have been sufficient during the index admission. Furthermore, the possibility of performing venous thrombectomy alongside the myotomy could be considered for the prompt resolution of venous thrombosis.

In summary, this case underscores the potential of PAES as a rare etiology of DVT, emphasizing the importance of considering it in the differential diagnosis of venous diseases in younger patients lacking common predisposing factors. Moreover, if CT imaging is available, meticulous evaluation of anatomical anomalies should be considered in young patients presenting with unprovoked DVT.

## Data Availability

The raw data supporting the conclusions of this article will be made available by the authors, without undue reservation.
